# Asylums in India

**Published:** 1894-04-14

**Authors:** 


### ASYLUMS IN INDIA.

### V.—THE PUNJAB.

The Lahore Asylum has a capacity for 296 patients, calculated at 100 superficial feet per patient, and the Delhi Asylum can accommodate 150 patients, estimating 36 superficial feet as sufficient for each patient. No explanation is given of the different basis for these two calculations. The movement of population at the two asylums was briefly as follows: 317 lunatics remained on January 1st and 115 were admitted during the year, giving a total population of 432, with a daily average strength of 328. Of these, 41 were discharged cured, 10 improved, 5 not-improved, 1 "otherwise," 1 escaped, and 34 died, thus leaving 340 under treatment at the close of the year. The percentage of cures to daily average strength works out at 11·44 at Lahore as compared with 14·94 in 1890, and at 15·19 at Delhi as compared with 20·43 in 1890. The death-rate fell at Lahore from 11·53 to 9·32, and rose at Delhi from 7·52 to 13·02. An epidemic of influenza accounted for six deaths in this latter institution, and there were also four deaths from pneumonia, unconnected with influenza. This large number of cases in a comparatively small community leads to the suspicion that the cause was a preventible local one.

The total expenditure under all heads was Rs.40,758 7a. 3p. at Lahore, and Rs.10,622 7a. 3p. at Delhi, and the total cost per head, including medicines, but excluding expenditure by the Public Works Department, was Rs.99 3a. 9p. at Lahore, and Rs.96 8a. 11p. In the asylums of the North-Western Provinces the amount rarely exceeds Rs.70, and is generally much lower, but taking it at the highest the excess expended in the Punjab asylums represents 28 per cent. The Inspector-General, however, is of opinion that these asylums cannot be managed so economically as those in the North-Western Provinces, the conditions being somewhat different. At the same time a nearer approach to them might be made.

